# Sustainable Vineyard Management with On-Field UV-C Irradiation: Impacts of Supplementary Applications on Grape Composition and Secondary Metabolites

**DOI:** 10.3390/plants15020298

**Published:** 2026-01-19

**Authors:** Claudio D’Onofrio, Giacomo Palai, Vincenzo Tosi, Daniele Ghidotti, Carmine Mattia Verosimile, Alessio Neri

**Affiliations:** 1 Department of Agriculture, Food and Environment, University of Pisa, Via del Borghetto 80, 56124 Pisa, Italy; giacomo.palai@unipi.it (G.P.); vincenzo.tosi@phd.unipi.it (V.T.); mattia.verosimile@phd.unipi.it (C.M.V.); 2 Nutraceuticals and Food for Health—Nutrafood, University of Pisa, Via del Borghetto 80, 56124 Pisa, Italy; 3 Tenuta dell’Ornellaia, Località Ornellaia 191, Castagneto Carducci, 57022 Livorno, Italy; d.ghidotti@studenti.unipi.it (D.G.); alessio.neri@ornellaia.it (A.N.)

**Keywords:** anthocyanin, aroma compounds, phenylpropanoid, *Vitis vinifera* L., VOCs

## Abstract

Research for sustainable viticulture practices has fostered interest in ultraviolet-C (UV-C) radiation as non-chemical tool for vineyard pathogen control; however, little information is available on their potential elicitation of berry metabolites. This two-year study investigated the impact of supplementary in-field UV-C applications, in addition to the vineyard sanitary protocols, on berry composition in Cabernet Sauvignon grapevines. In both experimental years, vegetative, yield, and berry technological parameters were determined at harvest, but they were not altered by UV-C treatments. Significantly higher concentrations of berry secondary metabolites were measured at harvest trough GC-MS and HPLC. UV-C treated vines had higher berry anthocyanins, particularly tri-hydroxylated forms (malvidin, delphinidin, petunidin), and flavonol concentrations (quercetin, myricetin derivatives), improving the potential for wine color stability and copigmentation. Glycosylated berry aroma compounds were also increased in UV-C vines, particularly some monoterpenes (geraniol, nerol, citronellol), C_13_-norisoprenoids (β-damascenone, β-ionone, 3-oxo-α-ionol), and volatile phenols (eugenol, 4-vinyl-guaiacol). These results highlighted the potential of UV-C in-field applications, in addition to pest management control, to increase grape quality traits by modulating berry phenolic and aroma profile without affecting productivity.

## 1. Introduction

Ultraviolet (UV) radiation represents the part of the electromagnetic spectrum between visible light and X-rays. Depending on wavelength, it is divided into UV-A (320–400 nm), UV-B (280–320 nm), and UV-C (200–280 nm). While UV-A and UV-B are natural components of solar radiation in the troposphere, UV-C is largely filtered by the stratospheric ozone layer.

In agriculture, interest in UV radiation has recently risen due to the need for sustainable alternatives to chemical pesticides. In fact, artificial UV-C irradiation has long been exploited for its germicidal activity, since absorption by nucleic acids causes DNA damage and inactivation of microorganisms [1]. In viticulture, sustainable crop protection is particularly relevant because of the high level of pesticides required, particularly to control fungal pathogens such as powdery mildew and downy mildew. In this light, UV-C irradiation has been proposed as a physical elicitor that can be easily integrated into vineyard management with limited risks of residues and environmental impact [2]. Experimental evidence confirms that UV-C treatments can reduce fungal infections in several crops, including tomato, lettuce, strawberry, and apple [3,4,5,6]. In grapevine, the available studies have shown significant reductions in powdery mildew and downy mildew incidence and severity following UV-C exposure, with effects persisting over time [7].

Beyond antimicrobial action, UV-C irradiation interacts with plant physiology and metabolism in complex ways. At the cellular level chloroplasts and mitochondria are particularly sensitive: inhibition of photosynthesis has been linked to UV damage of thylakoid membranes and photosystem II [8]. The development of reactive oxygen species under UV stress can also act as a signaling mechanism to promote plant defense responses [9], including the upregulation of many key enzymes of the berry secondary metabolites biosynthesis. Grapevine naturally synthesizes a wide range of secondary metabolites covering key roles in photoprotection, stress adaptation, and berry quality. Among these, phenylpropanoids constitute the main group induced by UV exposure [10]. Flavonoids, such as anthocyanins, flavonols, and proanthocyanidins accumulated in berry skins after UV exposure, protecting tissues from oxidative stress [11]. In fact, their biosynthesis is regulated by photoreceptors and transcription factors, including the *UVR8* signaling pathway, *MYB* genes, and flavonol synthases, particularly sensitive to light stimuli [12].

The UV radiation also influences the biosynthesis of berry terpenes and norisoprenoids, which contribute to grape and wine aroma. Several studies have demonstrated that UV-B radiation can enhance the accumulation of specific terpenoids in grape tissues. An experiment on vine leaves revealed that moderate UV-B exposure increased volatile terpenoid concentrations, suggesting an adaptive metabolic response as a stress-related change [13]. The timing and intensity of UV exposure are also critical. Reducing the natural UV radiation at different phenological stages, it was observed that the exposure during the ripening phase influenced the accumulation of free monoterpenes and sesquiterpenes in Shiraz grape berries [14]), highlighting the key role of both the berry developmental stage and the wavelength of UV. Recently, Yin et al. [15] observed that UV exclusion during berry ripening significantly decreased the concentration of several key aroma compounds, particularly monoterpenes. In Pinot noir, the combined effects of UV-B radiation and water deficit conditions altered the berry amino acid profile, which in turn affected the synthesis of aroma-active compounds during fermentation [16]. However, to the best of our knowledge, very little information is available on the effect of in-field UV-C application on grape quality; rather, the majority of the studies so far have investigated the UV-C as a post-harvest technique to enhance grape storability. Considering the potential of UV-C light to stimulate secondary metabolism, it is worth evaluating its impact in vineyards, under in-field real conditions. Some research has focused on the potential of UV-C against pathogens, but less explored is whether in-field UV-C supplementary applications within a phytosanitary protocol can simultaneously improve grape quality. The objective of this study was therefore to investigate the effects of vineyard applications of UV-C radiation on Cabernet Sauvignon grapes, delivered through supplementary treatments with respect to the defense protocol against fungal pathogens, to evaluate the potential beneficial effects on grape quality.

## 2. Results

### 2.1. Climatic Conditions and Vine Phenology

**Figure 1:**
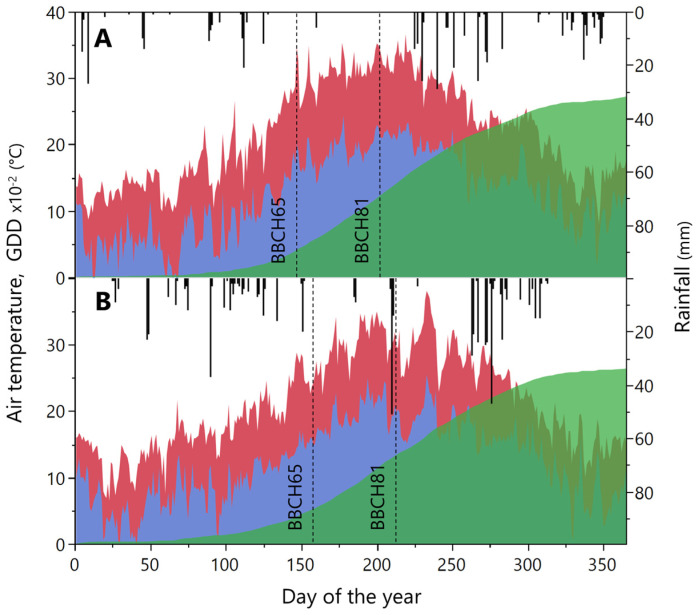
Maximum (red area) and minimum (blue area) air temperature. GDD × 10^−2^ (green area) and rainfall (vertical bars) measured at experimental site in 2022 (**A**) and 2023 (**B**). Vertical dotted lines indicate the phenological stages 65 and 81 according to the BBCH scale.

2022 was marked by high temperatures since mid-May (on May 26, a daily maximum temperature of 36.8 °C was recorded), which reports a monthly average of the daily maximum temperature of 25.8 °C (Figure 1).

The mean air temperatures of June (24.69 °C), July (26.47 °C), and August (25.89 °C) showed a prolonged period of high temperatures concomitantly with low rainfall. In 2023 the spring was mild with alternating rainfall in May (49 mm) and mild temperatures (average max 22.8 °C) (Figure 1). July and August were hot (average Tmax of 31.4 °C and 30.4 °C, respectively), with a peak of 38.1 °C in August, and the rainfall was concentrated in a few events during August (42 mm). Vine phenology in both years was affected by climatic conditions (Figure 1). In particular, the higher temperatures and low rainfall of 2022 accelerated the vine’s growth and berry ripening anticipating full flowering (day of the year, DOY, 148 and 155 in 2022 and 2023, respectively) and veraison (DOY 210 and 216 in 2022 and 2023, respectively) date, as well as the harvest date, even though the growing degree days accumulated at flowering and veraison were similar in both years (450 and 446 °C, 1369 and 1383 °C in 2022 and 2023, respectively) (Figure 1).

### 2.2. Vegetative Parameters and Fruit Yield

**Figure 2:**
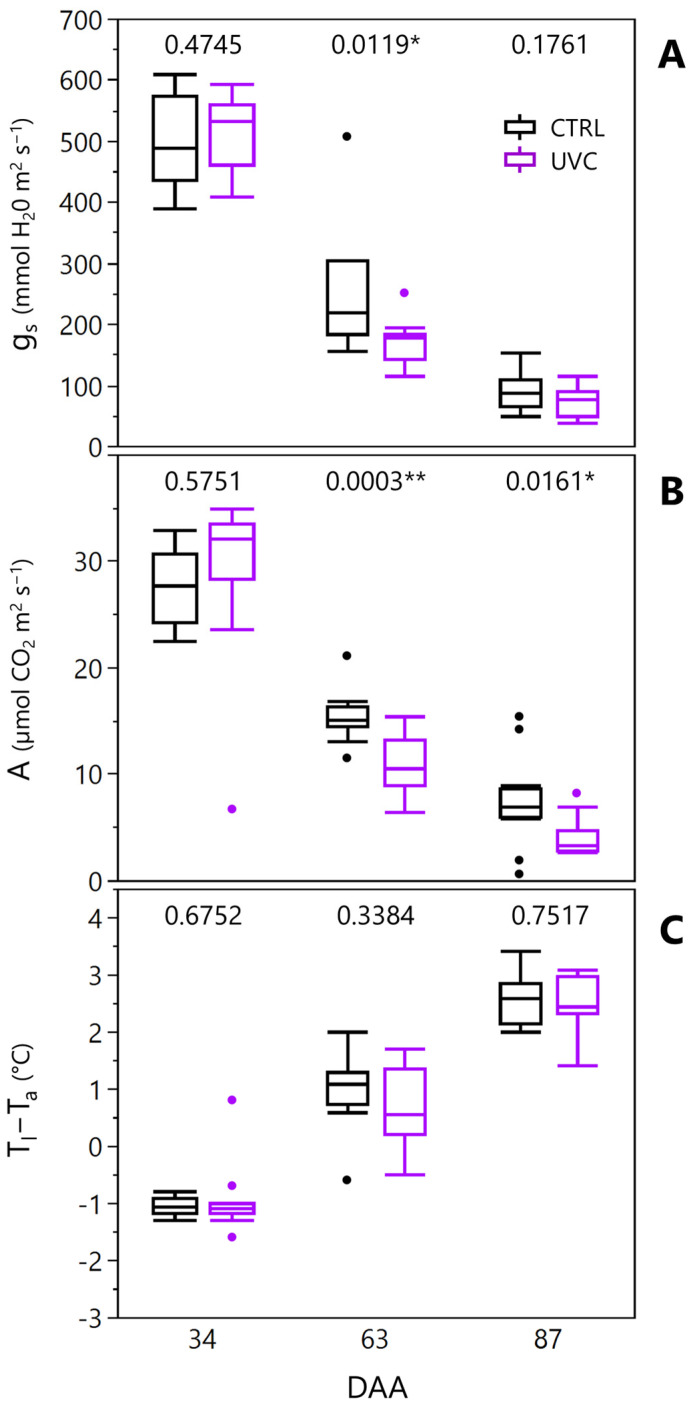
Stomatal conductance (**A**), net photosynthesis (**B**), and leaf blade to air temperature differential (**C**) measured on the experimental vines in 2023 at three different dates (34, 63, and 87 days after anthesis, DAA). *p*-values after t-student are reported for each date within the graph (* *p* < 0.05; **, *p* < 0.01).

The number of shoots per vine ranged from 7.60 to 8.77 and did not show significant differences between UV-C and CTRL (Table 1). Similarly, the number of fruity shoots (7.07–7.37), total leaf area (TLA, 1.43–1.62 m^2^), stem water potential (Ψ_stem_, −0.70–−0.87 MPa), number of clusters (11.27–13.05), fruit yield per vine (0.84–1.12 kg), pruning weight (0.37–0.49 kg), and Ravaz index (2.29–2.46) showed no statistically significant differences between treatments. The leaf gas exchange parameters were measured in 2023 at three different dates (Figure 2).

Stomatal conductance (g_s_) had similar values between UV-C and CTRL vines at 34 and 87 days after anthesis (DAA), whereas at DAA 63 significant lower values were measured in UV-C with respect to CTRL (−16%). Significantly lower values in UV-C vines were measured for net photosynthesis (A) as well, at DAA 63 and 87 (−31% and −28%, respectively). No differences were observed in the thermal differential between leaf blade and air temperature, measured concomitantly with leaf gas exchange (Figure 2C).

### 2.3. Berry Technological Parameters

In 2022, berry total soluble solids (TSS) ranged from 23.60 to 24.60 °Brix (regardless of sampling time), with slightly lower values in UV-C treated berries compared to CTRL, though differences were not statistically significant (Table 2). Berry pH values were similar between treatments either at T1 and T2, as were titratable acidity (TA, 4.39–4.74 g L^−1^, regardless of sampling time) and berry fresh weight (FW, 0.86–0.94 g, regardless of sampling time), with no significant treatment effects (Table 2). In 2023, TSS values were slightly lower overall (22.9–23.3 °Brix, regardless of sampling time), with no significant differences between UV-C and CTRL berries. Similarly, pH ranged from 3.39 to 3.50, TA from 4.20 to 4.55 g L^−1^, and berry FW from 1.01 to 1.11 g, with no significant effects of the UV-C treatment (Table 2).

### 2.4. Berry Flavonoids Compounds

**Figure 3:**
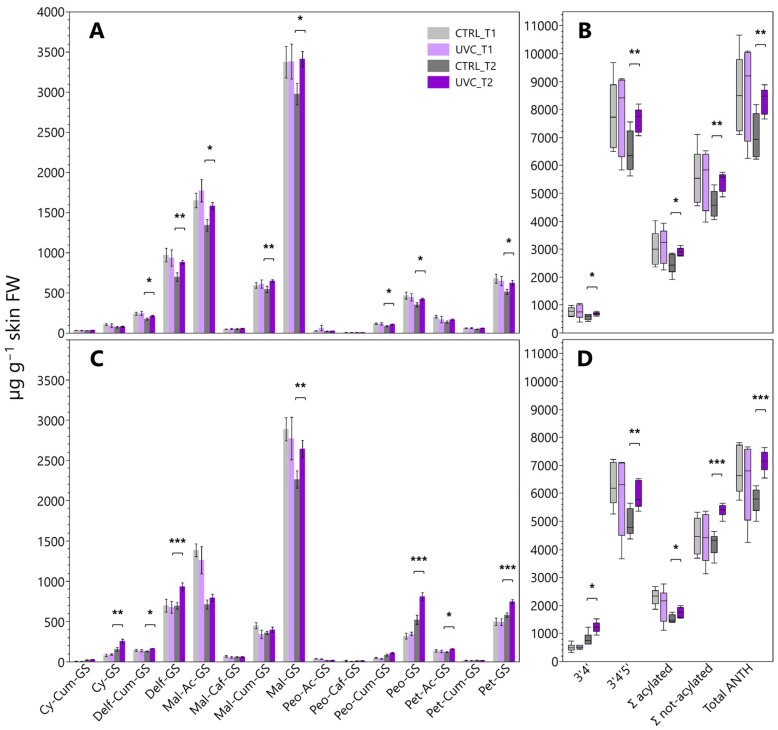
Skin anthocyanin concentration (µg g^−1^ berry skin fresh weight) measured at T1 and T2 in 2022 (**A**,**B**) and 2023 (**C**,**D**). Values are means ± standard deviation of six replicates per treatment. Asterisks indicate significant differences after t-student within each compound and sampling date. *, *p* < 0.05; **, *p* < 0.01; ***, *p* < 0.001. Legend: GS, glucoside; Cy, cyanidin; Delf, delphinidin; Mal, malvidin; Peo, peonidin; Pet, petunidin; Cum, cumaroyl; Ac, acetyl; Caf, caffeoyl.

The berry skin total anthocyanin concentration showed no significant differences between treatments at T1 in both years, while, at T2 the UV-C-treated berries had significant higher values compared to CTRL (Figure 3), with an increase of +18% in 2022 (8333.6 and 7061.3 μg g^−1^ in UV-C and CTRL, respectively) and +24% in 2023 (7140.8 and 5739.5 μg g^−1^ in UV-C and CTRL, respectively).

**Figure 4:**
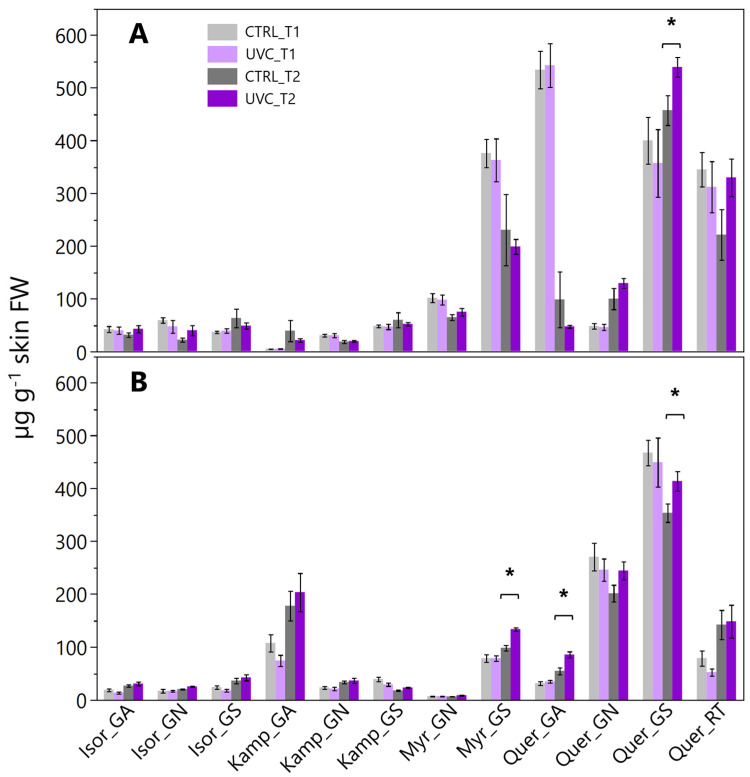
Skin flavonol concentration (µg g^−1^ berry skin fresh weight) measured on T1 and T2 in in 2022 (**A**) and 2023 (**B**). Values are means ± standard deviation of six replicates per treatment. Asterisks indicate significant differences after t-student within each compound and sampling date. *, *p* < 0.05. Legend: GS, glucoside; GA, galactoside, GN, glucuronide, RT, rutinoside; Isor, isorhamnetin; Kamp, kaempferol; Myr, myricetin; Quer, quercetin.

The 3′4′ and 3′4′5′ derivatives showed a similar pattern: in 2022, both types of compounds increased under UV-C at T2 (+19% and +18%, respectively), and it was even more pronounced at T2 in 2023 both for 3′4′ (+53% and +20%, respectively). Among single compounds, cyanidin glucoside and delphinidin glucoside showed significant increases under UV-C at T2 in 2023 (+66% and +34%, respectively), as did malvidin glucoside (+15% in 2022 and +17% in 2023) (Figure 3). Peonidin glucoside displayed a significant increase at T2 in 2023 (808.6 and 520.2 μg g^−1^ in UV-C and CTRL, respectively). The analysis of acylated vs. non-acylated anthocyanins revealed that UV-C consistently enhanced both fractions at T2. In 2022, Σ acylated increased from 2445.3 μg g^−1^ in CTRL to 2910 μg g^−1^ in UV-C, while Σ non-acylated showed smaller increases (+18%). In 2023, the effect was less pronounced for acylated forms but still evident in non-acylated ones (Figure 3). Total flavonol content did not differ significantly between UV-C and CTRL berries in both years and sampling times. Single compounds, however, showed variable responses depending on harvest time (Figure 4). In 2022, quercetin glucoside significantly increased at T2 in UV-C berries (539.7 and 457.7 µg g^−1^ in UV-C and CTRL, respectively), while other compounds did not show significant differences. In 2023, significant increases were observed in UV-C berries at T2 especially for myricetin glucoside (+36%), quercetin galactoside (+57%), and for quercetin glucoside (+17%) (Figure 4).

### 2.5. Berry Aroma Compounds

The berry-glycosylated volatile organic compounds (VOCs) were mainly affected by UV-C treatment at T1, whereas differences at T2 were less consistent (Table 3). Among compound classes, monoterpenes and C_13_-norisoprenoids had the most significant variations. Within monoterpenes, in 2022, at T1 UV-C significantly enhanced citronellol (+191%) with respect to CTRL, while nerol increased by +26%, and trans- and cis-8-hydroxylinalool by +38% and +47%, respectively. Similarly, α-citral (+29%) and geranic acid (+33%) were significantly higher in UV-C berries at T1. This pattern was confirmed in 2023, where UV-C treatment significantly increased nerol (+102%), α-citral (+88%), trans-8-hydroxylinalool (+45%), cis-8-hydroxylinalool (+40%), and geranic acid (+42%) at T1. Within the C_13_-norisoprenoids, 3-oxo-α-ionol consistently increased under UV-C at T1 in both seasons (+65% and +43% in 2022 and 2023, respectively). The 3-oxo-α-ionol derivatives also showed significant enhancement at T1 in 2023 (+39%, average between compounds). Benzene derivatives and aliphatic alcohols showed less variations, though methyl salicylate increased under UV-C at T1 in 2022 (+115%) and was significantly higher also in 2023, (+71%) and 3-hexen-1-ol was significantly higher under UV-C at T1 in 2022 (+54%). Also considering free VOCs, the main differences were observed at T1 with a reduced concentration of UV-C treated berries, especially in 2022, whereas in 2023 differences were largely non-significant (Table 4). Among aliphatic alcohols, 3-methyl-2-buten-1-ol and 1-octen-3-ol were reduced under UV-C in 2022 T1 (−83% and −75%, respectively). Phenolic compounds also significantly decreased under UV-C in 2022 T1, particularly guaiacol and 2,6-dimethoxyphenol (−31% and −41%, respectively). Similarly, several monoterpenes were reduced by UV-C at 2022 T1, including citronellol (−62%), 7-OH-geraniol (−63%), geranic acid (−48%), and 2,6-dimethyl-6OH-2,7-octadienoic acid (−57%), resulting in a decrease in total monoterpenes from 37.8 to 19 ng g^−1^. In contrast, in 2023 none of these compounds were significantly lower, and in some cases T2 values under UV-C were slightly higher than controls. Other classes of free VOCs, including vanillins and benzene derivatives, were not significantly affected by the treatment in either year.

## 3. Discussion

From a vegetative and productive point of view, the parameters measured on the experimental vines suggested that the supplementary UV-C applications did not interfere with vine growth and yield. Yield per vine, average cluster weight, pruning weight, and the Ravaz index were statistically similar between treatments, consistent with previous observations that supplementary UV field-treatments rarely affect grapevine productivity. In a recent experiment, Chardonnay grapevines subjected to night-time UV-C applications at 200 J m^−2^ across three consecutive seasons did not affect yield parameters [17]. Similar results were also reported in a field experiment on Chardonnay and on the interspecific hybrid Vignoles, using night-time UV-C applications [7]. Worth noting, these results demonstrated that the use of on-field UV-C treatments did not affect vine growth and yield, a key prerequisite for its potential adoption in vineyard protocols. In our experiments, the TSS concentration was not different between treatments, even though slightly lower values in UV-C treated berries were observed at both sample dates in 2022, a particularly stressful year characterized by low rainfall and high temperatures. It is plausible that we did not measure any significant differences because of the treatments protocol, consisting of only two supplementary UV-C applications during ripening. In Malbec grapevine, a significant effect of UV-B light exposure to increase berry sugar concentration was observed [18], even though sugar accumulation acceleration and reduced harvest concentration after UV-B application were reported as well [19]. These contrasting findings highlight that the impact of UV radiation on berry sugar accumulation is influenced not only by genotype but also by the specific experimental conditions, including whether experiments were performed under controlled vs. open-field conditions, the type of radiation applied (UV-A, UV-B, or UV-C), the total dose and frequency of application, and the phenological timing of irradiation exposure. Neither berry TA nor pH varied between treatments in both sampling times and years. In agreement, repeated UV-B treatments during ripening (daily 4–6 h exposures) applied on field-grown Tempranillo had no significant impact on berry titratable acidity [20]. Similar results were reported in Pinot Noir subjected to field UV exclusion or transmission [21], albeit another study reported decreased TA and increased pH in Shiraz berries under UV-screening with UV-B and UV-A filters during mid-to-late ripening [14].

The main hypothesis behind our experiment was that the additional application of UV-C during berry ripening may stimulate secondary metabolites biosynthesis, potentially increasing the overall grape quality. Many studies on tree fruit crops reported that UV radiation can enhance anthocyanin biosynthesis, including apple [22], peach [23], blueberry [24], and strawberry [25]. The effects on grape berries, however, remain variable. Higher anthocyanins concentrations were reported in Malbec and Tempranillo subjected to different on-field UV applications [20,26], and similar results were observed in different grapevine varieties even when subjected to UV-B after harvest [27]. A key factor to consider is the wavelength and the timing of exposure. Many experiments addressed UV-A or UV-B, while UV-C remains scarcely studied. Most of the literature investigating UV-C exposure in grapevines refers to post-harvest treatments, consistently reporting increased berry anthocyanin concentrations, often accompanied by the upregulation of key genes of the phenylpropanoid pathway [28,29,30]. From our results, grapes subjected to supplementary UV-C treatments during veraison and ripening had higher anthocyanin concentrations at both sampling dates. In particular we measured significant increment in malvidin, petunidin, and delphinidin 3-O-glucosides. Tri-substituted anthocyanins consistently increased in UV-C berries, with statistical significance at T2. Acylated anthocyanins, including malvidin 3-O-acetyl-glucoside and malvidin 3-O-p-coumaroyl-glucoside, also increased. Notably, the shift in the proportion of acylated and non-acylated forms that we observed suggested that UV-C may alter the balance between acylated and non-acylated anthocyanins, that, from an oenological point of view, could impact wine color stability and copigmentation potential. Berry total flavonol concentration at harvest was significantly higher in UV-C-treated grapes. Quercetin and myricetin derivatives were particularly enhanced, such as quercetin 3-O-glucoside, quercetin 3-O-rutinoside, and myricetin 3-O-glucoside. These results were consistent with the literature suggesting flavonols as reliable markers of UV exposure [31,32], as well as the literature showing significant increment under UV-B supplementary application in different cultivars and tissues, including leaves and berries [20,33,34].

Berry VOCs accumulation generally peaked before full phenolic maturity, consistently with the known temporal dynamics of aroma metabolism [35]. At T2, the VOCs concentrations had declined compared to T1, where UV-C berries showed higher concentrations of many different VOCs both in the glycosylated and free form. Notably, at the T1 stage, when treatment-induced differences were most pronounced, a distinct shift in the ratio of free-to-glycosylated VOCs was observed. In particular, UV-C treated berries exhibited a higher concentration of glycosylated forms alongside a concomitant reduction in free VOCs compared to the control. This inverse relationship suggests that the UV-C treatment may primarily modulate the glycosylation pathway, potentially driving the conversion of free volatiles into their conjugated forms, rather than acting as a direct trigger for de novo biosynthesis. Aliphatic alcohols were slightly affected, in agreement with Gil et al. [36], who investigated Malbec vines subjected UV-B supplementation from pre-flowering to harvest (0.40 W m^–2^). The authors observed that C6 alcohols, such as hexanol and 3-hexen-1-ol, were inconsistently affected by UV-B, even though a significant increase in terpenoid accumulation was observed as well, indicating a differential metabolic sensitivity of VOCs classes. In agreement, our data highlighted that monoterpenes tended to increase, with geraniol, citronellol, nerol, and limonene showing higher levels under UV-C. To the best of our knowledge, no information are available on the effect of on-field UV-C on berry terpenes; however, a wide literature has reported that monoterpenes often increase under different UV supplementary application [14,21,36]. Sasaki et al. [37], covering with UV-block Sauvignon Blanc berries from veraison to harvest, observed that the linalool content significantly decreased, as well as the expression levels of the genes involved in linalool biosynthesis. More recently, Miao et al. [14] demonstrated that supplemental UV-B irradiation on Cabernet Sauvignon clusters (0.3–0.45 W·m^−2^, daily application from veraison to harvest) consistently upregulated terpene synthases genes expression, leading to higher concentration of free and bound monoterpenes. In our experiment, the berry C_13_-norisoprenoids was a sensitive responsive volatile class to UV-C treatment, with significant increases in β-damascenone, β-ionone, and 3-oxo-α-ionol. Their enhancement under UV-C could also affect the typical varietal aroma, particularly since β-damascenone could mask herbaceous methoxypyrazines [38]. C_13_-norisoprenoids are produced through the oxidative cleavage of carotenoids and subsequent non-enzymatic rearrangements, and their accumulation is related to the pool of carotenoid precursors, which are known as sensitive to light conditions [39,40]. The higher glycosylated C_13_-norisoprenoid concentrations that we observed under UV-C exposure may reflect a reprogramming of carotenoid oxidative cleavage. Joubert et al. [41] observed that Sauvignon Banc berries exposed to UV-B excluding sheets accumulated higher norisoprenoid concentrations, particularly increasing β-cyclocitral. In contrast, no significant differences were observed in β-damascenone and β-ionone concentration in Pinot noir wines obtained from grapes subjected to transmitted or blocked UV radiation [21]. To the best of our knowledge, no information is available on the effect of on-field UV treatment on volatile phenols; however, our results highlighted their increment in UV-C berries, especially 4-vinyl-guaiacol, eugenol, and coniferol, contributing spicy and balsamic flavors while also participating in stable pyranoanthocyanin formation with malvidin.

## 4. Materials and Methods

### 4.1. Experimental Site

**Figure 5:**
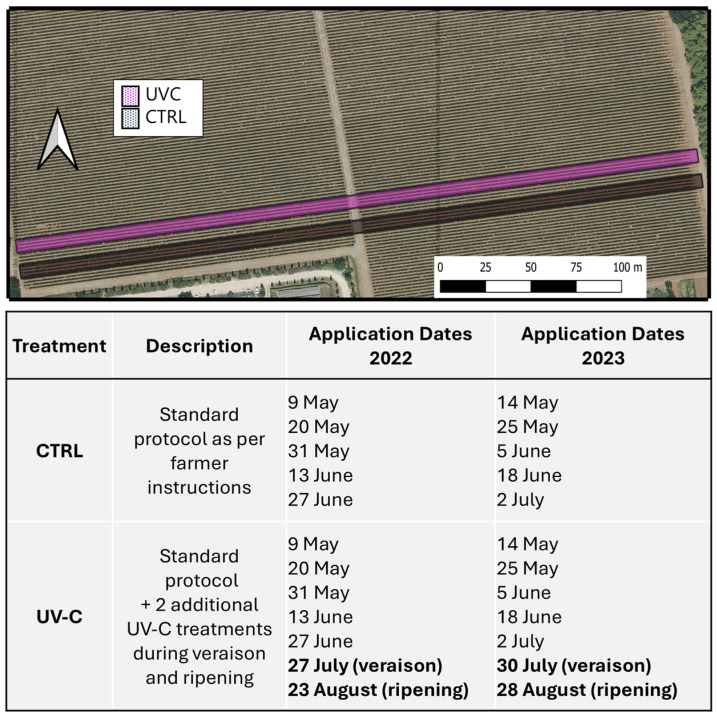
Experimental site and design and UV-C treatment protocol carried out in 2022 and 2023.

The experiment was carried out at the Ornellaia wine estate within the Bolgheri denomination, in Tuscany, Italy (43.219770° N, 10.589321° E), on Cabernet Sauvignon vines clone 337 (*Vitis vinifera* L.) grafted on 101-14 rootstock and planted in 2013. The vineyard was located at an altitude of 29 m a.s.l. with a planting distance of 2 × 0.8 m (6250 vines/hectare) and east/west row orientation (Figure 5).

The vines were trained according to a Guyot pruning system characterized by five–six buds on the cane and two buds on the spur. After bud break, the shoots were carefully selected and spatially settled. Three mechanical trimming operations were carried out between June and August with a knife trimmer. The soil profile was hydromorphic, calcareous in depth and skeletal, well-drained, and neutral (pH 7.1) on the upper part. It had a sandy clay loam texture with percentages of sand and clay between 61–66% and 26–30%, respectively. The soil was also characterized by a low quantity of organic matter (1.47%) and nitrogen as well as organic carbon. Soil management was characterized by spontaneous grassing in alternate rows. The grass cover is shredded and left to cover the ground, while the tilled inter-row was plowed on a monthly basis. The under-row was managed by alternating filling and working with an inter-row blade. Fertilization was carried out with a basal soil application in late winter supplying 40 kg N ha^−1^, 25 kg P_2_O_5_ ha^−1^, and 50 kg K_2_O ha^−1^ to maintain vegetative–reproductive balance. A foliar micronutrient treatment containing boron and zinc was applied at pre-flowering to prevent potential deficiencies and support reproductive processes. The meteorological data of the year were collected by a weather station located in the vineyard (Netsens s.r.l., Calenzano, Italy).

### 4.2. UV-C Treatments

The experimental design included the farm’s standard UV-C protocol developed for phytosanitary purposes (only UV-C treatments without any application of agrochemical products), which served as the control (CTRL), and a treatment (UV-C) which consisted of two additional UV-C treatments as an extension to those provided to the CTRL vines (Figure 5). These supplemental treatments were applied after the conclusion of those of the phytosanitary UV-C protocol, one at full veraison and the other one at an intermediate ripening stage (16 °Brix) (Figure 5). The UV-C light was emitted by a system composed of 10 UV-C lamps (95 W each, 254 nm), delivering a dose of 800 J m^−2^s^−1^ in field conditions. The lamps were mounted onto a towed bilateral machine, consisting of a load-bearing structure with hydraulically adjustable width and four panels (one pair per side), and adjustable according to the thickness of the vegetation, allowing passage 5–10 cm from the leaf wall during treatment. The tractor’s forward speed was kept constant at 0.8 m s^−1^. CTRL and UV-C–treated vines were randomly selected within two adjacent vineyard rows (each 185 m long). The CTRL rows were separated from the UV-C rows by six buffer rows to avoid cross-exposure. For both treatments, six replicates were established, each consisting of seven vines

### 4.3. Vines Vegetative Growth and Yield Parameters

The TLA per vine was determined at veraison on 18 vines per treatment. For each experimental vine, the number of main shoots and leaves, the number of lateral shoots and leaves, and shoots with clusters and the number of clusters were recorded. A random sample of 100 main leaves and 50 lateral leaves was collected, and the area of each leaf was measured (ImageJ, National Institutes of Health, Bethesda, MD, USA): the average area of main leaves and of lateral leaves was then calculated and used to scale up to vine level. The clusters from each vine were weighed at harvest, as well as the wood pruned in winter: the Ravaz index was then obtained as the ratio between fruit yield and the wood pruned weight. Leaf gas exchange parameters were measured on fully expanded leaves of 15 vines per treatment using a portable open system CIRAS-2 (PP Systems, Amesbury, MA, USA) according to the protocol reported in Palai et al. [42]. Measurements were taken on cloudless days between 9:00 a.m. and 11:00 a.m. at photosynthetic photon flux density greater than 1000 μmol·m^−2^·s^−1^, ambient CO_2_ ranging from 410 to 425 μL·L^−1^, and air temperature of 29.1 ± 0.9 °C (mean ± standard deviation). Vine water status was determined at veraison in both years by measuring stem water potential. The leaf was enclosed for at least 45 min in a non-transpiring shaded bag to block transpiration and then sampled to determine Ψ_stem_ once the potential reached equilibrium with the xylem according to the detailed protocol described in Tosi et al. [43]. A summary of all the measurements and analysis carried out during the two experimental years is reported in Table 5.

### 4.4. Berry Characteristics and Must Parameters

Two sampling dates were established to determine berry technological characteristics: T1, prior to the farm’s harvest date and empirically evaluated as coinciding with the peak of berry aroma accumulation (29 August and 6 September in 2022 and 2023, respectively), and T2, coinciding with the farm’s harvest date (13 and 26 September in 2022 and 2023, respectively). In both dates, six replicates per treatment of 50 berries each were collected and immediately taken to the lab to determine berry FW, TSS, pH, and TA. Berry weight was determined by weighing with a scientific scale. The must was obtained by pressing the grapes of and filtering. A digital refractometer (DBRwine, HM Digital Ltd., Seoul, Republic of Korea) was used to measure TSS (°Brix). The pH was measured with a pH meter (Hanna Instruments, Woonsocket, RI, USA), and TA was determined through titration using a graduated burette and a 0.1 N NaOH solution until the point of change to pH 8.2.

### 4.5. Berry Secondary Metabolites Determination

A further sample of 150 berries per replicate was collected at T1 and T2 to determine berry flavonoids and glycosylated VOCs. The protocol proposed by Downey and Rochford [44] was followed for the analysis of anthocyanins and flavonols using an aliquot of 50 berries per replicate. Using a scalpel and forceps, the skins were carefully separated from the pulp and crushed to powder in liquid nitrogen. An aliquot of 0.1000 ± 0.0005 g of powder was extracted with 2.0 mL of 50% (*v*/*v*) methanol in water on a rocking belt at 500 rpm for 20 min. The samples were then centrifuged (15 min at 13,000 rpm thermostated at 4 °C), and a portion of 100 µL of supernatant was transferred to vials for HPLC analysis. The injection volume for each analysis was 20 µL. For the separations, 10% formic acid in water (solvent A) was used with a 10% aqueous solution of methanol (solvent B) at a flow rate of 2.0 mL/min. The column temperature was maintained at 40 °C throughout the analysis. A Wakosil C-18 SS column (150 mm × 4.6 mm, 3 m packing; SGE, Ringwood, Australia) with an SGE C-18 guard column was used. Samples were run on an Agilent infinity 1260 HPLC system (Agilent, Mulgrave, Australia). Gradient conditions were as follows: 0 min, 18% B; 14 min, 29% B; 16 min, 32% B; 18 min, 41% B; 18.1 min, 30% B; 29 min, 41% B; 32 min, 50% B; 34.5 min, 100% B; 35–38 min, 18% B.

The berry glycosylated VOCs were determined following the protocol described in detail in D’Onofrio et al. [45] through solid phase extraction. Briefly, on 100 berries per replicate the skins were separated from the pulps. The skins were added with 20 mL of methanol and after one hour were re-aggregated with the pulp and 200 mL of tartaric buffer at pH 3.2. The sample was then centrifuged twice and added to 200 mg of pectolytic enzyme and 200 µg of internal standard (1-heptanol). The extract was then passed into C18 cartridges for the solid phase extraction, following the passages fully described in D’Onofrio et al. [45]. Chromatographic analysis was carried out using a GC-MS (Agilent 5975C mass spectrometer coupled to the Agilent 7890 Gas Chromatograph, both from Agilent, Santa Clara, CA, USA) with a fused silica capillary column (HP-INNOVAX), polar, 30 m long, with an internal diameter of 0.25 mm and a film thickness of 0.25 μm. The following experimental conditions were set for the analyses: helium flow rate 1 mL/min; splitless injection at 30 °C with an injection time of two minutes; injector temperature of 250 °C; interface temperature of 230 °C; temperature program of 30 °C for two minutes, from 30 °C to 60 °C with a rate of 30 °C min^−1^, from 60 °C to 90 °C with a rate of 2 °C min^−1^, from 190 °C to 230 °C with a rate of 5 °C min^−1^; and 15 min of isotherm. The identification of aroma compounds was carried out by comparing retention times and mass spectra with authentic standards or on the basis of indices and spectra reported in the literature. The quantitative determination was performed by comparing the peak area of each compound with that of the internal standard. The monoterpenes geraniol, linalool, and a-terpineol derivatives were grouped following the aggregation based on their common biosynthetic derivation as proposed by D’Onofrio et al. [45] with some revisions suggested by Palai et al. [46].

### 4.6. Statistical Analysis

All data were tested for normality and homoscedasticity through Shapiro–Wilk and Levene’s test. Differences between treatments were assessed using parametric Student’s t-tests for pairwise comparisons within each year and sampling date. All statistical analyses were performed using JMP Pro software (version 17.1, SAS Institute Inc., Cary, NC, USA).

## 5. Conclusions

This study demonstrated that vineyard supplementary UV-C applications during berry ripening, in addition to the phytosanitary protocol, could potentially increase grape quality without affecting vine vegetative–productive parameters. Supplementary UV-C application tends to reduce TSS accumulation in seasons with high vegetative stress on the vine, but increases berry anthocyanin and flavonol concentration, especially tri-hydroxylated forms that may improve wine color stability and copigmentation. Also, several berry volatile compounds increased in vines subjected to supplementary UV-C, particularly those belonging to monoterpenes, C_13_-norisoprenoids, and volatile phenols, highlighting the necessity and value of evaluating the potential impact on the resulting wine sensory profiles in future studies. These results confirm that UV-C radiation is an elicitor of secondary metabolism in grapes, even though the different outcomes reported among studies indicate that responses depend on cultivar, UV dose, timing, and environmental context. The integration of additional UV-C treatments into common UV phytosanitary protocols for vineyards could improve grape and wine quality, albeit further research should confirm these results also with other treatment protocols in long-term and multi-season experiments.

## Figures and Tables

**Table 1:** Mean value ± standard deviation of the vegetative-productive parameters measured on the experimental vines. *p*-values after t-student within each year are reported.

Year	Treatment	Ψ_stem_ (MPa)	TLA (m^2^)	Shoots (n)	Fruity Shoots (n)	Clusters (n)	Fruit Yield per Vine (kg)	Pruning Weight (kg)	Ravaz Index
2022	CTRL	−0.82 ± 0.13	1.51 ± 0.31	7.60 ± 1.33	7.07 ± 1.34	11.27 ± 2.70	0.84 ± 0.29	0.37 ± 0.13	2.36 ± 0.68
	UV-C	−0.87 ± 0.10	1.48 ± 0.44	7.63 ± 1.40	7.37 ± 1.50	12.53 ± 3.25	0.97 ± 0.40	0.42 ± 0.13	2.46 ± 1.02
	*p*	0.5210	0.3247	0.9250	0.4163	0.1059	0.1416	0.1561	0.6567
2023	CTRL	−0.75 ± 0.08	1.62 ± 0.29	8.77 ± 0.85	7.33 ± 0.75	12.76 ± 1.81	1.12 ± 0.58	0.49 ± 0.09	2.29 ± 0.77
	UV-C	−0.70 ± 0.15	1.43 ± 0.26	8.10 ± 0.71	7.27 ± 0.94	13.05 ± 2.05	1.03 ± 0.44	0.42 ± 0.11	2.45 ± 0.95
	*p*	0.6650	0.1863	0.8865	0.4921	0.3729	0.7825	0.6699	0.3228

**Table 2:** Mean value ± standard deviation of berry technological parameters measured at T1 and T2 in the two experimental years. *p*-values after t-student within each year are reported.

Year	Harvest Time	Treatment	TSS (°Brix)	pH	TA (g L^−1^ tart. Acid)	Berry FW (g)
2022	T1	CTRL	24.25 ± 1.09	3.41 ± 0.08	4.74 ± 0.31	0.86 ± 0.04
	(29 August)	UV-C	23.60 ± 0.73	3.46 ± 0.09	4.63 ± 0.24	0.89 ± 0.05
		*p*	0.1893	0.2966	0.4943	0.2503
	T2	CTRL	24.60 ± 0.91	3.52 ± 0.09	4.39 ± 0.36	0.93 ± 0.05
	(13 September)	UV-C	24.02 ± 0.66	3.55 ± 0.14	4.40 ± 0.4	0.94 ± 0.09
		*p*	0.2307	0.6213	0.9557	0.7960
2023	T1	CTRL	22.90 ± 1.05	3.39 ± 0.09	4.55 ± 0.35	1.01 ± 0.06
	(06 September)	UV-C	22.95 ± 0.78	3.50 ± 0.09	4.35 ± 0.33	1.05 ± 0.05
		*p*	0.7368	0.2548	0.1488	0.8125
	T2	CTRL	23.10 ± 1.12	3.42 ± 0.10	4.20 ± 0.32	1.11 ± 0.06
	(26 September)	UV-C	23.25 ± 0.69	3.49 ± 0.11	4.25 ± 0.31	1.08 ± 0.06
		*p*	0.7778	0.5497	0.4361	0.6999

**Table 3:** Glycosylated berry VOCs measured at T1 and T2 in 2022 and 2023. Values (ng g^−1^) are means of six replicates for each treatment. Different letters indicate significant differences between treatments after t-student within each year and sampling date. *, *p* < 0.05; **, *p* < 0.01; ***, *p* < 0.001; *n.s.* (not significant). The superscript letter on monoterpenes indicates the biosynthetic origin: ^G^, geraniol; ^L^, linalool; ^T^, α-terpineol.

	2022						2023					
	T1			T2			T1			T2		
	CTRL	UV-C	*p*	CTRL	UV-C	*p*	CTRL	UV-C	*p*	CTRL	UV-C	*p*
isoamyl alcohol	22.1	18.3	*n.s.*	25.3	30.5	*n.s.*	39.8	37.3	*n.s.*	76.8	63.7	*n.s.*
1-pentanol	8.4	7.5	*n.s.*	7.3	8.5	*n.s.*	13	14.1	*n.s.*	13.4	14	*n.s.*
3-methyl-2-buten-1-ol	14.7	14.5	*n.s.*	13.1	18.4	***	25	29.3	*n.s.*	28.3	35.4	*
1-hexanol	44.5	40.2	*n.s.*	53.5	51.1	*n.s.*	85.3	79.8	*n.s.*	95.1	83.7	*n.s.*
*cis*-3-hexenol	1	1	*n.s.*	1.3	1.3	*n.s.*	3.8	2.8	*n.s.*	3.4	3.1	*n.s.*
*trans*-2-hexenol	7.3	6.8	*n.s.*	6.6	6.3	*n.s.*	15.9	17	*n.s.*	9.3	10.5	*n.s.*
1-octen-3-ol	5.9	6	***	7.1	8	*n.s.*	18.9	20.2	*n.s.*	11.1	13.1	*n.s.*
1-octanol	3.6	7.2	*n.s.*	3.2	3.1	*n.s.*	5.1	5.3	*n.s.*	4.5	4.6	*n.s.*
(*E*)-2-octen-1-ol	2.4	2.7	*	1.6	1.5	*n.s.*	2	1.9	*n.s.*	2.1	1.8	*n.s.*
4-methyl-3-pentenol	1.4	2	*n.s.*	1.3	1.4	*n.s.*	2.2	4	*n.s.*	2.9	2.8	*n.s.*
Total aliphatic alcohols	111.3	106.2		120.3	130.1		211	211.7		247	232.8	
benzaldehyde	2.7	1.6	*n.s.*	1.6	1.6	*n.s.*	5.2	5.3	*n.s.*	16.7	7.8	*n.s.*
acetophenone	0.3	0.3	*n.s.*	0.4	0.3	*n.s.*	0.8	0.8	*n.s.*	0.8	0.7	*n.s.*
methyl salicylate	0.8	1.3	**	0.7	1.2	*n.s.*	2.9	4.2	*n.s.*	3.4	4.6	*n.s.*
benzyl alcohol	363.5	364.7	*n.s.*	257.9	314.1	*n.s.*	542	589.3	*n.s.*	457.7	476	*n.s.*
2-phenylethanol	532.5	558.8	*n.s.*	477.5	463.9	*n.s.*	832.4	812.6	*n.s.*	908.4	783.7	*
benzenepropanol	0.6	0.4	*n.s.*	1	1.1	*n.s.*	0.8	0.8	*n.s.*	0.7	0.8	*n.s.*
p-cymen-7-ol	2.5	2.8	*n.s.*	2.1	1.7	*n.s.*	4	3.9	*n.s.*	3.4	3.1	*n.s.*
6-methoxy-3-methylbenzofuran	2.8	2.9	*n.s.*	2.6	1.8	*n.s.*	4.5	5.6	*n.s.*	6.3	6.3	*n.s.*
3′,5′-dimethoxyacetophenone	7.5	10.1	**	6	5.9	*n.s.*	9.6	12	*n.s.*	11.1	10.1	*n.s.*
3,4-dimethoxybenzyl alcohol	9.1	21	*n.s.*	11.6	10.1	*n.s.*	11.3	12.2	*n.s.*	10.7	10.5	*n.s.*
2,3,4-trimethoxybenzyl alcohol	21.3	31.7	***	19.2	20.1	*n.s.*	36.7	38.7	*n.s.*	33	34.4	*n.s.*
3,5-dimethoxy-4-hydroxyphenylacetic acid	8.6	13.1	*n.s.*	6.9	7.2	*n.s.*	17.4	14	*n.s.*	9.3	11.7	*n.s.*
Total benzene derivatives	952.4	1008.9		787.5	828.9		1467.8	1499.5		1461.7	1349.7	
1-phenylethanol	2.1	2.1	*n.s.*	1.9	1.8	*n.s.*	3.9	3.9	*n.s.*	4.3	3.8	*n.s.*
guaiacol	11.5	12.4	*n.s.*	8.1	8.3	*n.s.*	4.7	13.5	*n.s.*	4.5	8.8	*n.s.*
eugenol	1.7	1.9	*n.s.*	1.3	1.3	*n.s.*	2.9	3.1	*n.s.*	2.7	2.9	*n.s.*
4-vinylguaiacol	522.1	849.5	**	463.2	505.6	*n.s.*	1047.7	1106.9	*	1044.1	898.2	*n.s.*
γ-hydroxyeugenol	6.4	8.9	*	5.9	6.3	*n.s.*	11.1	10.5	*n.s.*	11.1	10.4	*n.s.*
phenol-3,4,5-trimethoxy	31.4	34.2	*n.s.*	26.1	28.7	*n.s.*	46.3	51.3	*n.s.*	56.3	50.9	*n.s.*
Total phenols	575.2	909.2		506.5	552.1		1116.6	1189.3		1123.1	975	
methyl vanillate	9.1	21	*n.s.*	11.6	10.1	*n.s.*	11.3	12.2	*n.s.*	10.7	10.5	*n.s.*
acetovanillone	10	9.8	*n.s.*	9.3	8.4	*n.s.*	11.6	12	*n.s.*	7.5	7.5	*n.s.*
zingerone	7	9.2	*n.s.*	5.9	4.6	*n.s.*	12.4	11.4	*n.s.*	9.9	8.6	*n.s.*
homovanillic alcohol	10.4	12.3	*n.s.*	5.4	5.4	*n.s.*	18.9	17.1	*n.s.*	11.6	12.2	*n.s.*
3,4,5-trimethoxybenzyl-methyl-ether	24	68.8	*n.s.*	34.8	31.5	*n.s.*	21.4	23.1	*n.s.*	28.7	30.9	*n.s.*
homovanillic acid	75.2	86.4	*n.s.*	41.7	43.2	*n.s.*	64.9	78.6	*n.s.*	59.4	76.2	*n.s.*
acetosyringone	28.1	38.7	**	25	23.1	*n.s.*	53.7	52.7	*n.s.*	51.6	44.8	*n.s.*
Total vanillins	163.7	246.1		133.7	126.3		194.2	207.2		179.3	190.8	
(*E*)-furanoid linalool ox. A ^L^	3.4	3.2	*n.s.*	2.7	2.1	*n.s.*	3.7	3.6	*n.s.*	3.2	2.8	*n.s.*
(*Z*)-furanoid linalool ox. B ^L^	3	3.2	*n.s.*	2.5	2.2	*n.s.*	4.4	4.3	*n.s.*	3.8	3.4	*n.s.*
terpinen-4-ol ^T^	0.4	0.6	**	0.3	0.3	*n.s.*	1	1	*n.s.*	0.8	0.6	*n.s.*
ocimenol	10.7	12.4	*n.s.*	8	5.6	*	1.2	2.3	*n.s.*	0.8	1	*n.s.*
α-terpineol ^T^	12.7	14.8	*	9.5	6.6	*	10.4	13.1	*	9.2	9.1	*n.s.*
α-citral ^G^	0.7	0.9	*	0.4	0.5	*n.s.*	2.4	4.5	*.	3.3	3.3	*n.s.*
(*E*)-pyranoid linalool ox. C ^L^	5.9	5.8	*n.s.*	4.8	3.6	*n.s.*	5.3	4.5	*n.s.*	5	4	*n.s.*
(*Z*)-pyranoid linalool ox. D ^L^	1.8	1.8	*n.s.*	1.6	1.3	*n.s.*	2.4	1.9	*n.s.*	2.2	1.7	*n.s.*
citronellol ^G^	1.1	3.2	***	1	0.9	*n.s.*	11.4	10.2	*n.s.*	6.9	9.6	*n.s.*
lilac alcohol A ^L^	4.5	7.7	**	3.6	3.6	*n.s.*	13.5	13	*n.s.*	11.1	11.3	*n.s.*
nerol ^G^	2.3	2.9	***	1.9	1.8	*n.s.*	4.2	8.5	**	5	6.4	*n.s.*
geraniol ^G^	11.1	12.3	*n.s.*	7.6	7.3	*n.s.*	21.7	20.4	*n.s.*	16.7	16.1	*n.s.*
exo-2-hydroxycineole ^T^	13.7	13.1	*n.s.*	9.9	7.6	*n.s.*	12.7	12.9	*n.s.*	10.4	9	*n.s.*
2,6-dimethyl-3,7-octadiene-2,6-diol 1 ^L^	0.8	0.9	*n.s.*	0.3	0.3	*n.s.*	0.9	1.1	*n.s.*	0.6	0.7	*n.s.*
6,7-2OH-7-hydroxylinalool ^L^	1.3	1.9	*	1	0.9	*n.s.*	5.7	5.2	*n.s.*	2.9	3.4	*n.s.*
2,6-dimethyl-1,7-octadiene-3,6-diol 2 ^L^	2.3	3.1	*	1.1	1.4	*n.s.*	2.6	2.6	*n.s.*	3	2.2	*n.s.*
OH-citronellol ^G^	9.5	8.4	*n.s.*	6.2	4.9	*n.s.*	6.3	6.2	*n.s.*	5.5	4.6	*n.s.*
*trans*-8-hydroxylinalool ^L^	11.2	15.4	**	8.7	7.8	*n.s.*	21.3	30.9	*	16.6	16.3	*n.s.*
*cis*-8-hydroxylinalool ^L^	13.5	19.9	**	11.3	10.4	*n.s.*	21.8	30.5	*	17.2	16.9	*n.s.*
geranic acid ^G^	10.6	14.1	**	7.6	7.6	*n.s.*	14	19.9	**	13	11.9	*n.s.*
7-OH-α-terpineol ^T^	157.7	170.8	*n.s.*	99.7	74.3	*	166.1	174.1	*n.s.*	142.2	127.9	*n.s.*
2,6-dimethyl-6OH-2,7-octadienoic acid ^L^	30.6	37.7	*n.s.*	22.8	21.2	*n.s.*	39.2	40.4	*n.s.*	29.8	31.8	*n.s.*
Total monoterpenes	308.9	353.8		212.3	172.1		372.4	411.1		309.5	294.2	
actinidol A	1.3	1.3	*n.s.*	1	0.6	*n.s.*	1.2	1.1	*n.s.*	1.1	0.9	*n.s.*
actinidol B	2.2	2.1	*n.s.*	1.6	1.2	*n.s.*	2.2	1.9	*n.s.*	1.7	1.5	*n.s.*
3,4-dihydro-3-oxo-α-ionol (I)	17.6	21	*n.s.*	14.9	13.1	*n.s.*	24.8	25.4	*n.s.*	25.5	22.3	*n.s.*
3,4-dihydro-3-oxo-α-ionol (II)	33.6	40.8	**	30.4	25.9	*n.s.*	41.8	49.2	**	48.3	40.8	*n.s.*
3,4-dihydro-3-oxo-α-ionol (III)	44.9	55.3	*	43.5	35.4	*n.s.*	54.7	64.1	*	66.3	55.2	*n.s.*
3-oxo-α-damascone	19.6	22.7	*	19.1	18.3	*n.s.*	29.7	31.5	*n.s.*	33.1	26.8	*n.s.*
3-oxo-α-ionol	302.8	403.6	**	293.6	260.8	*n.s.*	442.2	513.6	**	502.4	450.1	*n.s.*
2,3-2OH-4-oxo-7,8-2OH-β-ionon	15.2	21.1	**	17.9	16.1	*n.s.*	21	21.1	*n.s.*	30.9	26	*n.s.*
7,8-dihydrovomifoliol	6.9	12.2	*	6.9	5.6	*n.s.*	10.4	10.5	*n.s.*	7.7	7.1	*n.s.*
Total C_13_-norisprenoids	444.2	580		428.8	377.1		628.1	718.4		717.1	630.6	
Total glycosylated VOCs	5016.5	6322.5		4270.6	4254.8		7870.2	8141.1		7845.1	7128.4	

**Table 4:** Free berry VOCs measured at T1 and T2 in 2022 and 2023. Values (ng g−1) are means of six replicates for each treatment. Different letters indicate significant differences between treatments after t-student within each year and sampling date. *, *p* < 0.05; **, *p* < 0.01; ***, *p* < 0.001; *n.s.* (not significant). The superscript letter on monoterpenes indicates the biosynthetic origin: ^G^, geraniol; ^L^, linalool; ^T^, α-terpineol.

	2022						2023					
	T1			T2			T1			T2		
	CTRL	UV-C	*p*	CTRL	UV-C	*p*	CTRL	UV-C	*p*	CTRL	UV-C	*p*
isoamyl alcohol	1.3	1.3	*n.s.*	1.4	0.6	*n.s.*	10.3	12.2	*n.s.*	15.8	11	*n.s.*
3-methyl-2-buten-1-ol	8.2	1.4	***	1.6	0.6	*	18.4	16.3	*n.s.*	6.3	9.7	*n.s.*
1-hexanol	60.7	50	*n.s.*	34.7	17.7	*n.s.*	172.9	219	*n.s.*	98	136.2	*n.s.*
*trans*-3-hexenol	2	1.7	*n.s.*	1.1	0.6	*n.s.*	8.9	10.8	*n.s.*	5.7	6.6	*n.s.*
*cis*-3-hexenol	20.3	22.1	*n.s.*	5.2	3.5	*n.s.*	54	67.3	*n.s.*	16.9	26.8	*n.s.*
*trans*-2-hexenol	175.4	148.7	*n.s.*	54.7	56	*n.s.*	600.5	583.7	*n.s.*	410.7	467.8	*n.s.*
1-octen-3-ol	11.8	3	***	2.6	1.1	*n.s.*	13.8	14.8	*n.s.*	3.8	4.4	*n.s.*
Total aliphatic alcohols	279.7	228.1		101.3	80.1		878.8	924		557.2	662.6	
benzaldehyde	0.3	0.2	**	0.2	0.1	*n.s.*	3.5	3.4	*n.s.*	3.4	2.4	*n.s.*
acetophenone	1.8	1.5	*n.s.*	0.7	0.4	*n.s.*	9.5	9.4	*n.s.*	5.8	4.6	*n.s.*
2,5-dimethyl-benzaldehyde	1.4	0.1	*n.s.*	0.3	0.1	*n.s.*	0.8	0.5	*n.s.*	0.8	0.6	*n.s.*
2-phenylethanol	52.3	36.7	*	16.7	19.7	*n.s.*	203.2	205.1	*n.s.*	193.9	173.4	*n.s.*
Total benzene derivatives	55.7	38.5		17.8	20.4		217	218.3		203.9	181	
guaiacol	3.9	2.7	**	2.1	1.2	*n.s.*	6.8	6.8	*n.s.*	6	4.4	*n.s.*
β-phenoxyethyl alcohol	23.4	26.7	*n.s.*	10.6	5.9	*n.s.*	72.4	66.3	*n.s.*	61.4	58.7	*n.s.*
4-vinylguaiacol	56.8	27	*n.s.*	37.2	18.9	*n.s.*	149.8	69.7	*n.s.*	132.1	90.7	*n.s.*
phenol-2,6-dimethoxy	43.4	25.4	**	14.8	16.7	*n.s.*	63.7	59.9	*n.s.*	69.3	55.5	*n.s.*
eugenol	0.4	0.4	*n.s.*	0.2	0.1	*n.s.*	1.9	1.9	*n.s.*	1.1	1.1	*n.s.*
phenol-3,4,5-trimethoxy	4.8	2.6	**	3	1.6	*n.s.*	17.5	16.1	*n.s.*	21.8	16.7	*n.s.*
Total phenols	132.8	84.7		67.9	44.4		312.1	220.6		291.6	227	
vanillin	1.7	1.2	*n.s.*	0.8	0.5	*n.s.*	6.3	4.9	*n.s.*	3.6	3.3	*n.s.*
methyl vanillate	26.8	21.6	*n.s.*	13.1	7.9	*n.s.*	90.7	98.5	*n.s.*	88.2	71.7	*n.s.*
acetovanillone	2.6	1.5	*	1.4	0.6	*n.s.*	12.8	12	*n.s.*	6.1	5.9	*n.s.*
homovanillic alcohol	3.4	2.7	*n.s.*	1.4	0.7	*n.s.*	10.4	13	*n.s.*	6.4	8.9	*n.s.*
homovanillic acid	10.3	12	*n.s.*	3.4	2	*n.s.*	50	46.4	*n.s.*	20.6	14.5	*n.s.*
acetosyringone	3.8	2.4	*n.s.*	1.8	0.9	*n.s.*	11.4	8.4	*n.s.*	11.4	10	*n.s.*
Total vanillins	48.5	41.3		21.8	12.5		181.7	183.2		136.3	114.3	
*p*-cymen-7-ol	0.5	0.3	*n.s.*	0.2	0.1	*n.s.*	1.7	1.4	*n.s.*	1.5	1.1	*n.s.*
citronellol ^G^	6.6	2.5	**	2.4	0.8	*n.s.*	21.4	19.3	*n.s.*	9.1	10.1	*n.s.*
exo-2-hydroxycineole	1.1	1	*n.s.*	0.4	0.3	*n.s.*	1.1	1.8	*n.s.*	1.1	1.4	*n.s.*
2,3-pinanediol	3.2	2.4	*n.s.*	1.1	0.6	*n.s.*	13.5	12.2	*n.s.*	10.7	8.9	*n.s.*
7-OH-geraniol ^G^	5.7	2.1	***	1.2	0.8	*n.s.*	6.6	7.9	*n.s.*	3.3	4.7	*n.s.*
*cis*-8-OH-linalool ^L^	2.7	2.2	*n.s.*	1.1	0.6	*n.s.*	15	10.5	*n.s.*	6.2	6.6	*n.s.*
geranic acid ^G^	7.7	4	**	2.2	1.2	*n.s.*	15.8	16.4	*n.s.*	10.7	9.4	*n.s.*
7-OH-α-terpineol ^T^	2.3	0.9	*n.s.*	2	0.7	*n.s.*	4.9	5	*n.s.*	4.5	3.3	*n.s.*
2,6-dimethyl-6OH-2,7-octadienoic acid ^L^	8.1	3.5	**	1.8	1.2	*n.s.*	77.7	43.1	*n.s.*	21.7	92.4	*n.s.*
Total monoterpenes	37.8	19		12.5	6.3		157.6	117.6		68.7	137.9	
Total Free VOCs	554.5	411.6		221.3	163.7		1747.2	1663.7		1257.7	1322.8	

**Table 5:** Summary of the measurements and analysis carried out during the two experimental years.

Year	Date	Measurements/Analysis
2022	29 July	TLA, Ψ_stem_
	29 August (T1)	Berry technological parameters (TSS, TA, pH)
		Berry flavonol and anthocyanin profile
		Berry free/glycosylated VOCs profile
	13 September (T2)	Vine vegetative-productive parameters
		Berry flavonol and anthocyanin profile
		Berry free/glycosylated VOCs profile
2023	7 July	Leaf gas exchange
	1 August	Leaf gas exchange
	4 August	TLA, Ψ_stem_
	25 August	Leaf gas exchange
	6 September (T1)	Berry technological parameters (TSS, TA, pH)
		Berry flavonol and anthocyanin profile
		Berry free/glycosylated VOCs profile
	26 September (T2)	Vine vegetative-productive parameters
		Berry flavonol and anthocyanin profile
		Berry free/glycosylated VOCs profile

## Data Availability

Data is contained within the article.
